# Improvements in pandemic preparedness in 8 Central American countries, 2008 - 2012

**DOI:** 10.1186/1472-6963-14-209

**Published:** 2014-05-09

**Authors:** Lucinda EA Johnson, Wilfrido Clará, Manoj Gambhir, Rafael Chacón- Fuentes, Carlos Marín-Correa, Jorge Jara, Percy Minaya, David Rodríguez, Natalia Blanco, Naomi Iihoshi, Maribel Orozco, Carmen Lange, Sergio Vinicio Pérez, Nydia Amador, Marc-Alain Widdowson, Ann C Moen, Eduardo Azziz-Baumgartner

**Affiliations:** 1 Influenza Division, National Center for Immunization and Respiratory Diseases, Centers for Disease Control and Prevention, 1600 Clifton Road, Atlanta, GA 30333, USA; 2 Influenza Program, Centers for Disease Control and Prevention Regional Office for Central America, San Salvador, El Salvador; 3 Department of Epidemiology and Preventive Medicine, Monash University, Melbourne, Australia; 4 Center for Health Studies, Universidad del Valle, Guatemala City, Guatemala; 5 Training of Epidemiology and Public Health Intervention Network, Atlanta, GA, USA; 6 General Directorate of Health Surveillance, Ministry of Health of El Salvador, San Salvador, El Salvador; 7 National Epidemiology Center, Ministry of Health of Guatemala, Guatemala City, Guatemala; 8 Council of Ministers of Health of Central America and the Dominican Republic, San Salvador, El Salvador; 9 Department of Epidemiology, Ministry of Health of Panama, Panama City, Panama; 10 General Directorate of Health Surveillance, Ministry of Health of Costa Rica, San José, Costa Rica

**Keywords:** Pandemic, Influenza, Preparedness, IHR, Central America, Capacity-building, Technical assistance

## Abstract

**Background:**

In view of ongoing pandemic threats such as the recent human cases of novel avian influenza A(H7N9) in China, it is important that all countries continue their preparedness efforts. Since 2006, Central American countries have received donor funding and technical assistance from the U.S. Centers for Disease Control and Prevention (CDC) to build and improve their capacity for influenza surveillance and pandemic preparedness. Our objective was to measure changes in pandemic preparedness in this region, and explore factors associated with these changes, using evaluations conducted between 2008 and 2012.

**Methods:**

Eight Central American countries scored their pandemic preparedness across 12 capabilities in 2008, 2010 and 2012, using a standardized tool developed by CDC. Scores were calculated by country and capability and compared between evaluation years using the Student’s *t*-test and Wilcoxon Rank Sum test, respectively. Virological data reported to WHO were used to assess changes in testing capacity between evaluation years. Linear regression was used to examine associations between scores, donor funding, technical assistance and WHO reporting.

**Results:**

All countries improved their pandemic preparedness between 2008 and 2012 and seven made statistically significant gains (p < 0.05). Increases in median scores were observed for all 12 capabilities over the same period and were statistically significant for eight of these (p < 0.05): country planning, communications, routine influenza surveillance, national respiratory disease surveillance, outbreak response, resources for containment, community interventions and health sector response. We found a positive association between preparedness scores and cumulative funding between 2006 and 2011 (R^2^ = 0.5, p < 0.01). The number of specimens reported to WHO from participating countries increased significantly from 5,551 (2008) to 18,172 (2012) (p < 0.01).

**Conclusions:**

Central America has made significant improvements in influenza pandemic preparedness between 2008 and 2012. U.S. donor funding and technical assistance provided to the region is likely to have contributed to the improvements we observed, although information on other sources of funding and support was unavailable to study. Gains are also likely the result of countries’ response to the 2009 influenza pandemic. Further research is required to determine the degree to which pandemic improvements are sustainable.

## Background

In 2006, the World Health Organization (WHO) published the revised International Health Regulations (IHR) (2005) in response to an increase in global travel and trade and the emergence and re-emergence of infectious diseases, such as avian influenza [1]. These regulations provided an updated legal framework to guide the international community in the prevention and mitigation of acute public health risks, including pandemic influenza. The revised IHR became legally binding in June 2007 with the expectation that Member States would implement the recommendations within five years. In the intervening period, the world experienced its first influenza pandemic in over 30 years with the global circulation of Influenza A (H1N1)pdm09 virus [2].

Low and middle-income countries face greater challenges in preparing for pandemic influenza than high-income countries [3] and, in the event of a pandemic, they are thought to be at risk of higher mortality rates [4,5]. Central America is a region of middle-income countries: Belize, Costa Rica, El Salvador, Guatemala, Honduras, Nicaragua and Panama. Since 2006, these seven countries, together with the Dominican Republic, have been the recipients of technical assistance and funding from the U.S. Centers for Disease Control and Prevention (CDC) to develop their influenza surveillance capacity and pandemic preparedness. In addition, the WHO has published a number of strategy and guidance documents to support countries in their efforts to develop and strengthen pandemic preparedness [5-10]. These documents include the Pan American Health Organization (PAHO) and CDC generic protocol for influenza surveillance, published in 2006 to specifically help countries in the Latin American region standardize their surveillance systems [10].

In 2008, CDC published an assessment tool to help countries determine the status of their influenza pandemic preparedness across twelve capabilities; functions, resources or activities identified as critical to pandemic preparedness and response [11]. WHO describes preparedness as the ability to detect zoonotic and human influenza viruses, respond to widespread disease should it occur, and minimize the impact of disease on the economy and society [6]. The content of the tool was developed during 2006 and 2007, in the context of WHO recommendations for national pandemic preparedness, including the IHR (2005), as well as the best available science and practice standards for preparedness at the time. The principle aim of the tool has been to help countries and their donor partners identify opportunities for improvement so that funding and technical assistance can be targeted to further develop and enhance pandemic preparedness.

The purpose of this study was to measure changes in pandemic preparedness in Central America using evaluations conducted in eight countries which took place in 2008, 2010 and 2012. We also explored factors associated with these changes including the 2009 influenza pandemic and, U.S. CDC technical assistance and funding provided to the region since 2006.

## Methods

This study was determined not to be human subjects’ research; data about a human that cannot be linked back to the human is exempt [12]. Our data sources included 1) information collected during evaluation of country programs which was in a manner that subjects cannot be identified and 2) information from existing, publicly available data sets.

### Study population

The countries in this study included: Belize, Costa Rica, the Dominican Republic, El Salvador, Guatemala, Honduras, Nicaragua and Panama. For the purposes of this paper, the terms ‘Central America’ and ‘Central American countries’ refer to these eight countries.

### Data collection tool

CDC’s *National Inventory of Core Capabilities for Pandemic Influenza Preparedness and Response Tool *[11] assesses 12 specific operational capabilities of pandemic preparedness: country planning, research and use of findings for pandemic influenza preparedness, communications, epidemiologic capability, laboratory capability, routine influenza surveillance, national respiratory disease surveillance and reporting, outbreak response, resources for containment, community-based interventions to prevent the spread of influenza, infection control, and health sector pandemic response. The titles for some of these capabilities have been truncated or adapted throughout the paper for practicality and clarity, for example, ‘research and use of findings for pandemic influenza preparedness’ is referred to as ‘research’.

Each capability is composed of four indicators which describe specific health activities or public health functions related to that capability. These indicators are measured on a scale of zero to three where zero corresponds to very limited capability, one represents a low level of capability, two corresponds to a moderate level of capability and three indicates an advanced level of preparedness. A short description defines the minimum requirements for attaining each performance level (0, 1, 2 or 3) and these requirements range from defining the simple presence or absence of a public health activity, resource or function to increasing levels of coverage, timeliness or quality for that indicator [11] (Additional file 1: Table S1). One of the four indicators for two capabilities in the tool, ‘country planning’ and ‘health sector pandemic response’, is a split-indicator which is composed of two components. Each component is scored as described for each indicator (i.e. 0, 1, 2 or 3) and the overall score for the split-indicator is calculated by averaging the two component scores.

### Data collection

Each country’s level of influenza pandemic preparedness was measured in 2008, 2010 and 2012. Implementation of the tool requires the participation of persons responsible for pandemic preparedness in a country; participants were identified by the ministry of health from each country in partnership with CDC. They included, among others: epidemiologists, influenza laboratory staff, risk communication experts, emergency and disaster response personnel, health-care providers, and animal and environmental health experts, as well as, partners such as WHO, the World Bank, the U.S. Agency for International Development and local non-government organizations. The self-assessment process was facilitated by a CDC representative and took place over one to two days. The 12 capabilities were discussed among the participants until a consensus was reached regarding the score to assign to each of the 48 corresponding indicators per country. If necessary, participants accessed country-specific data sources such as their national pandemic preparedness plans or laboratory reports, to assist with their decision-making. Prior to their participation, countries were informed that the evaluations were voluntary and that data collected through the tool would only be published anonymously or in aggregate.

### Pandemic tool data analyses

Scores for each capability were calculated by taking the sum of their four respective indicators. Pandemic preparedness scores for each country were calculated as a percentage by summing the scores for each of the 12 capabilities and dividing the totals by the maximum score possible (12 capabilities × 4 indicators × maximum indicator score of 3 = 144). Percentage scores by capability were determined by calculating the median score for the eight countries for each capability, and dividing by the maximum score possible for a capability (4 indicators × maximum indicator score of 3 = 12). Missing data were given a score of zero. Higher percentage scores denote greater levels of preparedness. Scores were compared across years using the Wilcoxon rank-sum test. Linear regression was used to explore associations between funding and pandemic preparedness scores. All statistical analysis was performed using Stata MP Version 11 (StataCorp LP, College Station, U.S.).

### Funding and technical assistance data analyses

We accessed data from the CDC-Central America Regional Office to determine the allocation of budgeted influenza and pandemic preparedness funds and provision of technical assistance to the eight countries between 2006 and 2012. We used linear regression to analyze the association between cumulative funding and pandemic preparedness scores for each country. To account for the likely delay between preparedness budgeting and expenditure, we regressed 2006-2007, 2006-2009 and 2006-2011 funding data against 2008, 2010 and 2012 scores, respectively. Technical assistance was calculated by summing the months of service provided by cooperative agreement-funded staff to each country, *pro rata*. We used linear regression to analyze the association between cumulative technical assistance and pandemic preparedness scores for each country; technical assistance up to and including the month prior to an evaluation was regressed against the score for each evaluation.

### WHO FluNet data analyses

We extracted data from the WHO influenza virological surveillance database, FluNet, to determine the number of influenza specimens reported from Central America between January 1, 2008 and December 31, 2012 [13]. The Wilcoxon Rank Sum test was used to compare changes in the number of specimens reported over time. Linear regressions were performed to establish the relationship between 2008 pandemic scores and FluNet specimens reported in 2007-8, 2009-10 and 2011-12, respectively, with the aim of evaluating the association between baseline scores and a measurable outcome of influenza-related activity (i.e. increased reporting being indicative of influenza testing capacity which is, in turn, representative of preparedness).

## Results

### Trends in pandemic preparedness between 2008 and 2012

**Figure 1:**
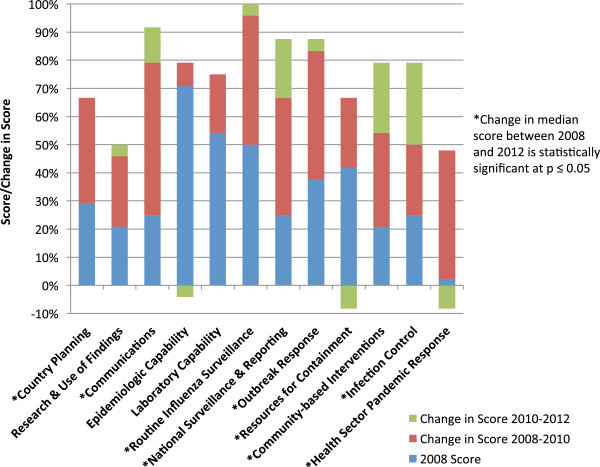
Median scores and changes in median scores for pandemic preparedness by capability combined for 8 Central American countries.

In 2008, countries scored epidemiology (median 71%, [IQR 63–79]), laboratory (median 54%, [50-83]) and routine influenza surveillance (median 50%, [42–69]) capabilities as most developed and community interventions (median 21%, [8–25]), research (median 21%, [8–58]), and health sector response (2%, [0–9]) capabilities as least developed (Figure 1, Table 1). The highest preparedness score for a country in 2008 was 59% and the lowest was 12%.

**Table 1:** Median influenza pandemic preparedness scores by capability for 2008, 2010 and 2012

**Pandemic capability**	**Median scores [IQR]**		
	**2008**	**2010**	**2012**
Country Planning	29% [19, 52]	67% [48,78]	67% [58,71]*
Research	21% [8, 58]	46% [42,63]	50% [25,77]
Communications	25% [19, 60]	79% [73,88]	92% [75,92]*
Epidemiology	71% [63, 79]	79% [67,85]	75% [63,77]
Laboratory	54% [50, 83]	75% [67,83]	75% [71,83]
Routine Surveillance	50% [42, 69]	96% [90,100]	100% [98,100]*
National Surveillance	25% [19, 42]	67% [58,77]	88% [81,94]*
Outbreak Response	38% [29, 50]	83% [79,100]	88% [73,94]*
Containment	42% [29, 50]	67% [58,77]	58% [56,73]*
Community Interventions	21% [8, 25]	54% [38,63]	79% [67,100]*
Infection Control	25% [17, 44]	50% [31,77]	79% [56,88]*
Health Sector Response	2% [0, 9]	48% [45,60]	40% [33,63]†

*Difference in median score between 2008 and 2012 is statistically significant at p ≤ 0.05 (Wilcoxon Rank Test).

**†**Difference in median score between 2008 and 2012 is statistically significant at p ≤ 0.01 (Wilcoxon Rank Test).

In 2012, countries scored routine influenza surveillance (median 100%, [IQR 98–100]), communications (median 92%, [75–92]), national respiratory disease surveillance (median 88%, [81-94]) and outbreak response (median 88%, [73–94]) capabilities as most developed and resources for containment (median 58%, [56–73], research (median 50%, [25–77]) and health sector response (median 40%, [33–63]) capabilities as least developed (Figure 1, Table 1). The highest preparedness score for a country in 2012 was 90% and the lowest was 59%.

Increases in median scores were observed for all 12 capabilities between 2008 and 2012 and were statistically significant for eight of these: country planning (p < 0.05), communications (p < 0.05), routine influenza surveillance (p < 0.05), national respiratory disease surveillance (<0.05), outbreak response (p < 0.05), resources for containment (p < 0.05), community interventions (p < 0.05) and health sector response (p < 0.01). Analyses by country showed a statistically significant increase in pandemic preparedness for seven of the eight countries (p < 0.05) between 2008 and 2012.

### Funding, technical assistance and pandemic preparedness

**Figure 2:**
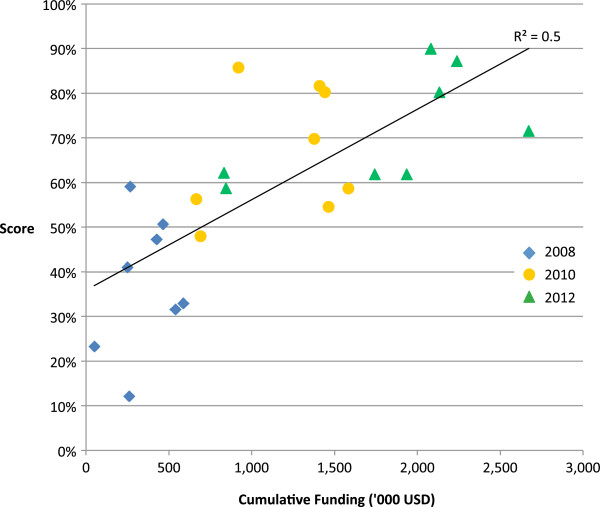
Pandemic preparedness scores 2008, 2010 and 2012 and cumulative funding received from 2006 to 2011.

From 2006 to 2011, the eight countries we studied were allocated a median of 335,000 [IQR 253,000-360,000] USD per year in cooperative agreement funding from CDC. This funding was distributed among surveillance activities (49%), laboratory activities (8%), research (18%) and other preparedness activities (26%). We found a positive association between pandemic scores in 2008, 2010 and 2012 and cumulative funding from 2006-2007, 2006-2009 and 2006-2011, respectively (R^2^ = 0.5 (i.e. 50% of the variance in scores was explained by cumulative funding) p < 0.01) (Figure 2). Each country has also benefitted from a median of 6.0 [5.7-6.9] person-years of technical assistance between January 2006 and April 2012. We found a positive association between pandemic preparedness scores and cumulative technical assistance (R^2^ = 0.2, p < 0.05) however, this association was no longer significant when funding was included in the regression; scores and funding remained significantly associated (R^2^ = 0.6, p < 0.001). Technical assistance and funding were themselves highly correlated (R^2^ = 0.6, p < 0.001).

### Laboratory capabilities in 2012

Between 2008 and 2012, two national laboratories were designated as WHO National Influenza Centres (NICs), bringing the total to six NICs in six countries for the region in 2012 (Table 2). The national laboratories of all eight countries utilize indirect fluorescent antibody (IFA) assays to detect influenza and seven have introduced quantitative reverse transcription polymerase chain reaction (qRT-PCR) testing since 2006 (Table 2). Seven countries use IFA testing to detect influenza at local hospitals, three of which also use qRT-PCR (Table 2). In 2012, six of the eight countries we studied were performing virus isolation and characterization (Table 2).

**Table 2:** Laboratory capabilities for identification of seasonal and pandemic influenza in Central America in 2012

**Country**	NIC* **(year designated)**	**IFA† National Lab**	**IFA Hospital Lab (# sites)**	**qRT-PCR‡ National Lab (year introduced)**	**RT-PCR Hospital Lab**	**Virus isolation**
Panama	yes (2007)	yes	Yes (2)	Yes (2007)	Yes (1)	Yes (1978)
Nicaragua	yes (2009)	yes	Yes (2)	Yes (2006)	No	Yes (2007)
Honduras	yes (2007)	yes	Yes (2)	Yes (2009)	No	Yes (2007)
Guatemala	yes (2009)	yes	Yes (3)	Yes (2009)	Yes (1)	Yes (2002)
El Salvador	yes (2005)	yes	Yes (3)	Yes (2009)	No	Yes (2003)
Dominican Republic	No	yes	Yes (1)	Yes (2009)	No	No
Costa Rica	yes (2006)	yes	Yes (3)	Yes (2008)	Yes (1)	Yes (2004)
Belize	No	yes	No (0)	No	No	No
**Total (Regional)**	**6**	**8**	**16**	**7**	**3**	**7**

*National Influenza Centre.

†Indirect Fluorescent Antibody Assay.

‡Quantitative reverse-transcription polymerase chain reaction.

### WHO FluNet reporting between 2008 and 2012

**Figure 3:**
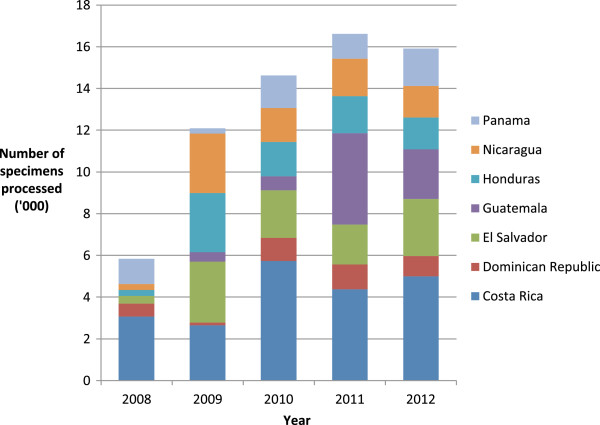
Number of specimens processed and reported to FluNet for seven Central American countries from January 1, 2008 to December 31, 2012.

**Figure 4:**
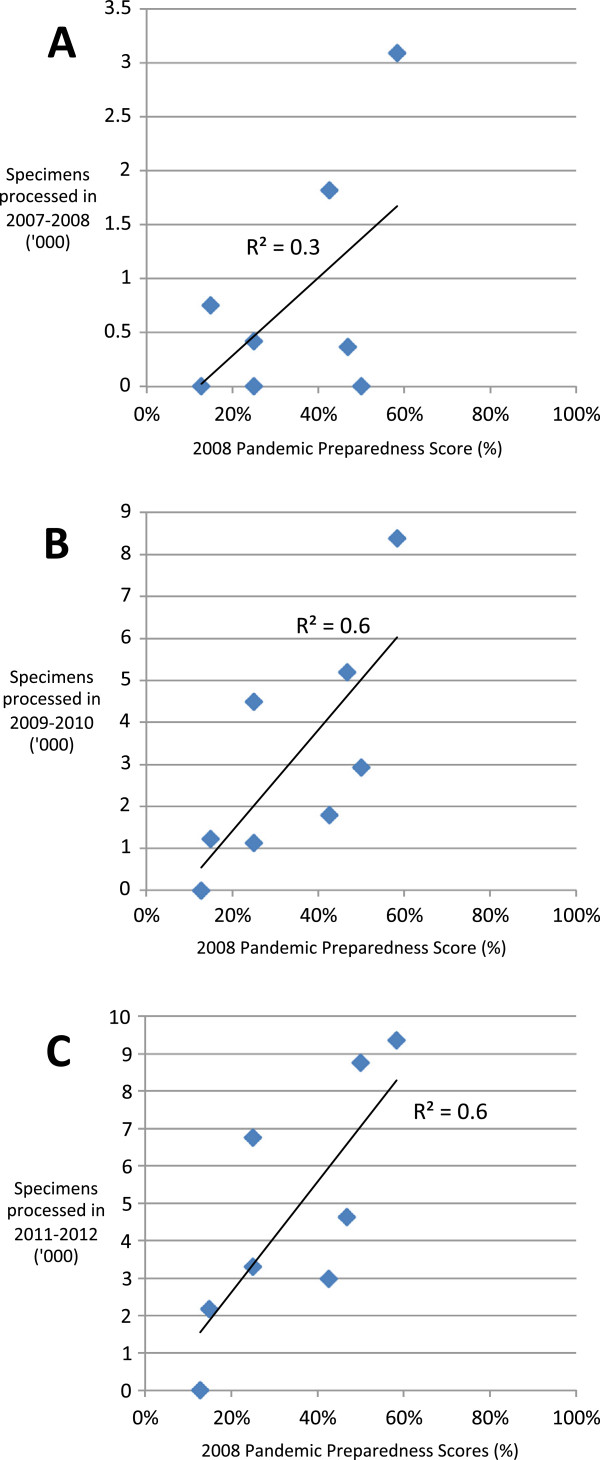
Pandemic preparedness scores in 2008 and specimens processed as reported to WHO FluNet in 2007 and 2008 (A), 2009 and 2010 (B), 2011 and 2012 (C), respectively.

In 2008, five countries reported processing 5,551 specimens (median 328, IQR 0–765) while in 2012, seven countries reported processing 18,172 specimens (median 2,089, [1,380–2,997]) (p < 0.01) (Figure 3). We found a positive association between baseline pandemic preparedness scores and FluNet specimens processed for 2007-8 (R^2^ = 0.3), 2009-10 (R^2^ = 0.6, p < 0.05) and 2011-12 (R^2^ = 0.6, p < 0.05), respectively (Figure 4).

## Discussion

Central America has made significant improvements in influenza pandemic preparedness between 2008 and 2012. In particular, we measured significant gains in the following areas determined to be critical to pandemic preparedness: country planning, national respiratory disease surveillance, resources for containment, communications, routine influenza surveillance, outbreak response, community-based interventions and health sector response.

Since 2006, U.S. CDC has provided funding to Central America for influenza surveillance and pandemic preparedness, which we found to be significantly associated with overall pandemic preparedness scores between 2008 and 2012. The greatest proportion of funding (49%) supported surveillance activities, which might, in part, explain the significant improvements we found in ‘routine influenza surveillance’ and ‘national respiratory disease surveillance’ capabilities between 2008 and 2012. Improvements in these capabilities reflect greater integration of epidemiological and virological data, an increase in sentinel sites, more timely data analysis and reporting, improved cross-notification between ministries of health and agriculture and heightened public awareness and reporting of severe respiratory illness.

While the improvement observed in ‘laboratory capability’ for the region was not statistically significant, the proportion of funding allocated to laboratory activities over the period was only 8%. Furthermore, the median baseline score for this capability was relatively high at 54% leaving less room for large improvements to be made. Nevertheless, the increase in reporting to FluNet (Figure 3), the expansion of influenza laboratory techniques (Table 2), and attainment of NIC status in two countries’ national laboratories between 2008 and 2012, is indicative of laboratory improvements taking place during the study period.

In the same period that Central America received funding from CDC, the region allocated a portion of this funding to hiring staff with expert knowledge in influenza, mostly epidemiologists, who were embedded in each country’s ministry of health. In addition, one staff member spent 40% of their time providing technical assistance across the region. Total technical assistance amounted to 5.7 person-years per country between 2006 and 2011 and included: training in epidemiological methods for surveillance and laboratory techniques (IFA, qRT-PCR and cell culture for virus isolation), data analysis and management, rapid response and preparedness, advice for establishing new sentinel sites as well as specific support to ministries of health and NICs during the 2009 pandemic.

Technical assistance was not significantly associated with preparedness scores when it was included as an explanatory variable with funding; however, our funding data included salaries for technical assistance. Since funding and technical assistance were found to be highly correlated, we suggest technical assistance is likely to have contributed to the improvement observed in overall pandemic preparedness in the region and that its explanatory effect has largely been accounted for by the funding variable.

The occurrence of the 2009 influenza pandemic is likely to have contributed to the gains made in pandemic preparedness capabilities during the study period since the greatest improvements in scores were observed between 2008 and 2010. During 2009 and 2010 countries executed their pandemic preparedness plans; they activated their rapid response teams, increased surveillance, laboratory testing and reporting, distributed public health messages through print media, radio and television and set-up call centers or public hotlines. They also mobilized anti-viral stockpiles where available, enhanced infection control procedures in hospitals and clinics and coordinated their response with other sectors: education, disaster preparedness and emergency response, police and military (Wilfrido Clará, personal communication, 12/03/13). While improvements in preparedness were likely precipitated by the 2009 pandemic response, increased reporting to FluNet prior to 2009 suggests that some improvements preceded the pandemic; in 2006, three of the countries we studied reported processing a total of 615 specimens to WHO FluNet which increased to five countries reporting a total of 5,551 by the end of 2008. FluNet reporting is indicative of a country’s influenza surveillance and laboratory capacity, including their ability to collect and test specimens for influenza. In addition, the step increase in the positive association between 2008 pandemic preparedness scores and specimens reported to FluNet prior to (2007-8) and following the pandemic (2009-2010, 2011-12) suggests that preparedness activities prior to the pandemic were, to some extent, predictive of a country’s ability to respond.

In 2012, countries scored their preparedness for research (50%), resources for containment (58%) and health sector response (40%) as least developed. These results may suggest the need for greater investments and longer time-frames to make progress in some capabilities over others. For example, to make improvements in the ‘health sector response’ capability, as defined by the tool, required greater surge capacity for human resources, hospital beds and ventilators, while improving ‘resources for containment’ required enhanced antiviral stockpiling and distribution. In low- and middle-income countries, there is typically a shortage of health care staff, hospital infrastructure is limited and often inadequate, and resources, such as ventilators and antivirals, are scarce [3,14]. To make improvements in the capability of ‘research’, required greater collaboration between human and animal health sectors, use of research data to inform preparedness decisions, the allocation of funding to address research priorities, and active scientific engagement, for example, through attendance at conferences and the publication of findings. Investment in research is low in Central America [15] while engagement with research communities and access to scientific knowledge is known to be limited in low and middle-income countries [16]. Further to this, research requires a highly skilled labour force which is a challenge where gross enrollment in tertiary education ranges between 15% in Guatemala and Honduras to 50% in Costa Rica and Panama, and where tertiary completion rates are low [17].

Prior studies describing the state of country or regional-level pandemic preparedness have used the WHO pandemic planning checklist to assess preparedness at a single point in time [7,18-20]. For example, in 2006, Central American countries used a modified version of the WHO checklist and assessed their preparedness lowest for ‘maintaining essential services’ and highest for ‘implementation, testing and revision of national plans’ [21]. A separate study in Latin America from 2008 which evaluated the completeness of national strategic plans also used the WHO checklist and found surveillance and communications were adequately addressed but the health care sector and public health interventions were not well-developed [20]. In 2008, we also found countries to be stronger in routine influenza surveillance (50%) and communications (25%) than in health sector response (2%).

There are some limitations to our study. First, we used budgeted funding data which may not represent final expenditure between different preparedness activities. Second, we cannot readily separate the effect of technical assistance from funding on pandemic preparedness scores because our funding data includes a large component of technical assistance, namely, staff salaries. Third, the countries we studied are likely to have received some funding and technical assistance from sources other than CDC, and may have mobilized national resources for preparedness, particularly during the 2009 pandemic; any additional funds and resources would also contribute to the gains we observed in preparedness over the period. Fourth, we report an association between funding and pandemic preparedness only; the study was not designed to establish causation. Fifth, it is difficult to measure the extent to which the 2009 pandemic response supported the improvements we observed in preparedness, particularly between 2008 and 2010. Finally, we have not compared the countries we studied with countries that were not funded by, and did not receive technical assistance from, CDC over the same period. While self-assessments may be vulnerable to responder bias, steps were taken to minimize this; a variety of people were involved from each country, primary data sources were used to inform decision making, and the rating levels for each indicator were mostly well defined.

While not statistically significant, we saw the erosion of gains made in some capabilities between 2010 and 2012 (Figure 1); further studies might explore factors which determine the sustainability of pandemic preparedness. The extent to which pandemic preparedness predicts health outcomes associated with influenza infection is difficult to measure where morbidity and mortality data is not routinely collected [5]. Additional research would be required to examine the impact of preparedness activities on influenza deaths and disabilities in Central America.

## Conclusions

Central American countries have made significant improvements in pandemic preparedness between 2008 and 2012. U.S. donor funding and technical assistance for influenza surveillance and pandemic preparedness, provided to the region since 2006 is likely to have contributed to these changes, suggesting that bilateral support for Central America in this area of public health has been important and successful. The gains in preparedness we observed may also be attributed to increased activity associated with the 2009 influenza pandemic although there is evidence that some improvements preceded this event. The participation of the region in WHO GISRS, as evidenced by increased reporting to FluNet and NIC attainment during the study period, demonstrates the commitment of Central American countries to the IHR (2005). While progress in pandemic preparedness has been made in Central America in recent years, there is a need to determine the degree to which these gains are sustainable and the impact they may have on influenza morbidity and mortality. In view of ongoing pandemic threats such as the recent human cases of novel avian influenza A(H7N9) in China, it is important that Central American countries continue to invest in pandemic preparedness activities.

## Competing interests

The authors declare that they have no competing interests.

## Authors’ contributions

LEAJ, MG, AM and EAB all contributed to the study design. WC, RC-F, CM, JJ, PM, DR, NB, NI, MO, CL, SVP and NA collected and provided the data used for the analysis. LEAJ, MG and EAB did the data analysis. LEAJ, WC, MG, MA, AM and EAB interpreted the data. LEAJ drafted the paper. All authors reviewed and approved the final version.

## Pre-publication history

The pre-publication history for this paper can be accessed here:

http://www.biomedcentral.com/1472-6963/14/209/prepub

## Supplementary Material

Additional file 1: Table S1 National Inventory of Core Capabilities for Pandemic Influenza Preparedness and Response. Click here for file
