# Supplementary material for: Improvements in pandemic preparedness in 8 Central American countries, 2008 - 2012
Source: BMC Health Serv Res. 2014 May 9;14:209. doi: 10.1186/1472-6963-14-209 (PMC4022548; doi:10.1186/1472-6963-14-209)
Supplement: Additional file 1: Table S1 — National Inventory of Core Capabilities for Pandemic Influenza Preparedness and Response. [file 1472-6963-14-209-S1.docx]

**Supplementary Table 1: National Inventory of Core Capabilities for Pandemic Influenza Preparedness and Response**

| **CAPABILITY** | **INDICATOR** | **LEVEL OF CAPABILITY** | | | |
| --- | --- | --- | --- | --- | --- |
|  |  | **0** | **1** | **2** | **3 (advanced)** |
| Capability 1: Country planning | (a) Status of plan | No complete plan | Draft plan includes essential minimum elements of preparedness as identified in the WHO checklist | Plan has been reviewed by all relevant sectors of the government | Plan has been adopted nationally and is updated routinely based on exercises or other emerging information |
|  | (b) Dissemination | Plan is not widely disseminated or available to the public | Plan is known, but not widely available | Plan is published and widely disseminated at national level | Plan published and widely available to multiple sectors, partners, sub-national levels and public. |
|  | (c) Exercises | No testing of plan or preparation for testing the plan | Preparation for testing the plan | Parts of plan tested through tabletop exercises | Plan tested through tabletop exercises with follow up actions to address deficiencies or enhance quality |
|  | (d) Coordination and resources for implementation of country plan | No clearly defined decision-making structure in place | Clearly defined decision- making structure in place at national level | National-level decision- making structure is multisectoral; standard operational procedures for essential functions developed | National decision-making structure engages other countries in region and World Health Organization (WHO) |
|  |  | No allocation of resources for work of country plan | Financial resources for work of country plan included in budget | Financial resources prioritized based on needs and pandemic phase | Mechanism for sustainability of financial resources for work of country plan |
| Capability 2: research and use of findings for pandemic influenza preparedness | (a) Collaboration between human and animal health | No or limited collaboration between human and animal health domains | Sessions on animal health at meetings on human health and vice-versa | Cross-discipline national scientific group(s) established; group has met at least once in past 12 months | Cross-discipline national scientific group produces recommendations, policies, shared research agenda, or similar written product. |
|  | (b) Research priorities | No or limited research priorities identified | Research priorities identified | Plan to address priority research activities | At least partial funding to address priority research activities |
|  | (c) Environment of support for research and use of findings | No or limited scientific exchange | Scientists engage in domestic and international exchange | Government participates actively in national influenza research agenda or research and development strategy | Government allocates financial resources for priority research and development activities |
|  | (d) Use of data to inform decisions for pandemic influenza preparedness | No or limited evidence of use of data or research findings | Mechanism established for communicating important findings to decision or policy makers | ≥1 decision in past 12 months based on data or research findings | ≥3 decisions in past 12 months, including ≥1 based on regional data or research findings. |
| Capability 3: Communications | (a) Status of communications plan | No or limited operational communications plan | Operational communications plan covers some, but not all phases of influenza pandemic | Operational communications plan covers all phases of influenza pandemic | Pre-pandemic phases of operational communications plan tested or implemented |
|  | (b) Messaging | No or limited content developed; no identification of target audiences | Communications materials tailored to target audiences and translated into country's major languages | Templates for communications materials developed by phases of influenza pandemic | Communications materials tested |
|  | (c) Dissemination | No or limited identification or use of formal and informal communication channels | Formal and informal communication channels identified and in use or tested at the national level | Formal and informal communication channels identified and in use or tested at sub-national level | Formal and informal communication channels functional; improved based on information from testing or use |
|  | (d) Staffing | No spokespersons or communications staff identified; Spokespersons and communications staff at the national level identified but not trained | Spokespersons and communications staff at the national level identified and trained | Spokespersons and communications staff at the sub-national level identified and trained | Spokespersons and communications staff participate in exercises or outbreak response |
| Capability 4: Epidemiologic capability | (a) Operational status | No or limited planning or preparation | Adequate staffing and resources for timely monitoring and making recommendations on population health status, disparities, and acute incidents at the national level | Adequate staffing and resources for timely monitoring and making of recommendations on population health status, disparities, and acute incidents at the sub- national level | Adequate staffing and resources for timely monitoring and making recommendations on population health status, disparities, and acute incidents at the local level |
|  | (b) Epidemiologists and field epidemiologists | ≤1 practicing public health epidemiologist per million population | >1 practicing public health epidemiologists per million population | ≥3 practicing public health epidemiologists per million population; ≥1 practicing public health epidemiologist per sub- national level in 75% of sub-national levels | >10 practicing public health epidemiologists per million population, plus epidemiologist vacancies 25% |
|  | (c) Quality of public health epidemiologists | No or limited discussion of competencies for public health epidemiologists | Critical competencies and/or standards of performance identified for public health epidemiologists | Explicit process exists for quality assurance and quality improvement among practicing epidemiologists | Roster of public health epidemiologists and observable process for strengthening the profession |
|  | (d) Training | No or limited training in applied epidemiology | Utilizes training program in other country, preparing for program in country, or informal training embedded in another program; current situation not meeting national needs | Some form of epidemiology training program established in country; duration of training less than one year; meets some national needs | Established, ongoing training program with dedicated resources; accredited; annual cohorts or graduates; production adequate to meet national needs |
| Capability 5: Laboratory capability | (a) National influenza laboratory network | No or limited planning for laboratory for testing of influenza | National laboratory for testing of influenza | National laboratory with one or more sub-national laboratories sending specimens for testing or confirmation | National laboratory that routinely returns results of testing to referring labs |
|  | (b) bio-saftey level (BSL) and routine testing of specimens | No or limited planning for laboratory for testing of influenza | National laboratory with bio-safety level 2; does not routinely test influenza specimens | National laboratory with bio-safety level 2; routinely tests influenza specimens; participates in WHO External Quality Assurance Project (EQAP) | National laboratory with bio-safety of at least level 3; able to isolate avian influenza in humans |
|  | (c) Methods | No testing or identify influenza virus using rapid tests | Identify seasonal influenza virus, type and sub-type; identify novel influenza viruses using molecular techniques | Isolate seasonal influenza virus, type and subtype using Hemagglutination Inhibition test | Full antigenic and genetic characterization of influenza viruses; isolate novel influenza viruses under bio-safety level 3+ |
|  | (d) Participation in WHO system | No or limited reporting to WHO; planning or preparation to comply with International Health Regulations (IHR) | Working towards fulfilling terms of reference for a National Influenza Center; regularly reports to WHO; shares specimens and/or isolates with WHO | Established National Influenza Center; actively reports through FluNet; routinely shares specimens and/or isolates for seasonal and avian influenza | Actively reports and shares results with WHO within 48 hours of laboratory confirmation of a potential Public Health Emergency of International Concern (PHEIC) |
| Capability 6: Routine influenza surveillance | (a) Integration of virologic and epidemiologic surveillance | No surveillance for severe, acute hospitalized respiratory infections or less severe outpatient managed respiratory disease | ≥1 site collecting virologic data on respiratory disease, either in the outpatient or inpatient setting | ≥1 sentinel site collecting virologic ***and*** epidemiologic data on both hospitalized ***and*** non-hospitalized respiratory disease | Multiple sentinel sites with sufficient geographic distribution to produce nationally representative epidemiologic and virologic data on both severe and mild respiratory disease |
|  | (b) Data publication | Data published or distributed <2 times per year | Data published or distributed ≥2 times per year | Data published or distributed >2 times per year but less than weekly during influenza season | Data published or distributed weekly during influenza season |
|  | (c) Timeliness | Data received, analyzed and distributed to relevant parties on time <20% of the time | Data received, analyzed and distributed to relevant parties on time 20 - 50% of the time | Data received, analyzed and distributed to relevant parties on time 51 - 80% of the time | Data received, analyzed and distributed to relevant parties on time >80% of the time |
|  | (d) Case definitions | No or limited preparation of case definitions | Draft case definitions prepared | Case definitions adopted, but not WHO case definitions | Standard WHO case definitions used |
| Capability 7: National respiratory disease surveillance and reporting | (a) Awareness of need to report | No or limited efforts to educate health care workers and public | Education campaign for health care workers implemented, but not comprehensive; awareness campaign for public implemented with some public health messages delivered to targeted audiences on broadcast media | Both education campaign for health care workers and awareness campaign for public expanded to wider audiences | Public awareness campaign expanded to national audience and multiple media forms; reporting mechanism for general public and health care workers functional; mechanism for sustainability exists |
|  | (b) Rumor reporting and media scanning | No or sporadic monitoring of media and other informal data sources | Program for monitoring media and other informal data sources implemented at national level; records maintained | Program for monitoring media and other informal data sources implemented at sub- national level; records maintained; regular communication among levels; ≥1 potential alert investigated per month | Complete records of response at national and sub-national level; ≥2 potential alerts investigated per month |
|  | (c) Cross-notification | No or limited cross- notification between ministries of health and agriculture | Cross-notification between ministries of health and agriculture, but sometimes delayed; communication mechanism not systematized | Timely, systematic cross- notification between ministries of health and agriculture at national level | Joint investigations of events |
|  | (d) Timeliness | Few recognized respiratory outbreaks of significance or clusters of severe disease reported within 48 hours of recognition | Some recognized respiratory outbreaks of significance or clusters of severe disease reported within 48 hours of recognition | Many of recognized respiratory outbreaks of significance or clusters of severe disease reported within 48 hours of recognition | Most of recognized respiratory outbreaks of significance or clusters of severe disease reported within 48 hours of recognition |
| Capability 8: Outbreak response | (a) Human resources for outbreak response | Ad hoc response | People for ≥1 team at national level who are trained in WHO rapid response protocol | People for ≥1 team at sub- national level who are trained in WHO rapid response protocol | People for ≥1 team at each sub-national level who are trained in WHO rapid response protocol; mechanism exists for sustainability |
|  | (b) Logistical resources for outbreak response | No or limited availability of equipment for investigation or initial response to novel influenza | Equipment is accessible and organized for response for ≥1 team at national level | Equipment is accessible and organized for response for ≥1 team at sub-national level | Equipment is accessible and organized for response for ≥1 team at each sub- national level |
|  | (c) Exercises or response | Ad hoc or irregular response to outbreaks or exercises | ≥1 team at national level responds or practices at least once per year | ≥1 team at sub-national level responds or practices at least once per year | ≥1 team at each sub- national level responds or practices at least once per year; central organization or authority coordinates response or exercises |
|  | (d) Activation of team | Response not fully organized, equipped, or delivered within 4 days of notification of potential Public Health Emergency of International Concern (PHEIC) | Trained, equipped team begins response to potential Public Health Emergency of International Concern (PHEIC) on site within 3 days | Trained, equipped team begins response to potential Public Health Emergency of International Concern (PHEIC) on site within 2 days | Trained, equipped team begins response to potential Public Health Emergency of International Concern (PHEIC) on site within 1 day and submits laboratory specimens within 24 hours, or results within 72 hours of investigation |
| Capability 9: Resources for containment | (a) Availability of antivirals | No or limited antivirals stored within country | Antivirals for 20-day containment involving 1200 persons | Antivirals for 20-day containment involving 12,000 persons | Antivirals for 20-day containment involving 100,000 persons |
|  | (b) Storage facilities | No government storage facilities for antivirals | Government storage facility exists, but not designed for storage of pharmaceuticals; insufficient security, temperature control, inventory tracking, rotation and refresh of stock | Government pharmaceutical storage facility exists with adequate security, temperature control, inventory tracking, and refresh of stock | Government has formal agreement with licensed pharmaceutical storage facility |
|  | (c) Exercises and practice | No activity in the past 12 months | Table top exercise (or similar) within past 12 months | Drill, simulation, or practice within past 12 months | Evidence of ongoing activity in this area |
|  | (d) Distribution of materials | Materials for containment can reach <25% of country’s geographic area within 24 hours | Materials for containment can reach ≥25% - 49% of country’s geographic area within 24 hours | Materials for containment can reach 50% - 74% of country’s geographic area within 24 hours | Materials for containment can reach ≥75% of country’s geographic area within 24 hours |
| Capability 10: Community-based interventions to prevent the spread of influenza | (a) Social distancing | No or limited planning or preparation | Some plans or guidance for closing/re-opening schools and workplaces and cancellation of public gatherings | Clear written criteria for closing/re-opening schools and workplaces and cancellation of public gatherings in pandemic phases 4-6 | Clear written criteria staged by phase of pandemic is publicly available and has been widely disseminated to schools, workplaces, and communities |
|  | (b) Critical infrastructure | No or limited planning or preparation for maintenance of essential services | Some plans or guidance for maintenance of essential services | Clear written criteria for maintenance of essential services in pandemic phases 4-6 | Clear written criteria staged by severity of pandemic has been widely disseminated to and tested by essential service providers |
|  | (c) Voluntary isolation and quarantine | No or limited planning or preparation | Some recommendations for voluntary isolation and quarantine | Some experience in applying voluntary isolation and quarantine combined with antiviral treatment or prophylaxis | Isolation and quarantine have been consistently applied early and are accepted by communities |
|  | (d) Percent of districts with plan | Planning or preparation | Written community intervention/mitigation plans in <5% of levels below sub-national | Written community intervention/mitigation plans in 5-25% of levels below sub-national | Written community intervention/mitigation plans in >25% of levels below sub-national |
| Capability 11: Infection control | (a) Standards of infection control by level of health care system | No or limited discussion of draft standards for infection control | Standards for infection control exist for central health facilities designated to care for suspect or confirmed cases infected with novel strains; standards meet or exceed WHO standards | Infection control standards exist for each level of health care system (national level, sub- national, and one level below sub-national); system for assessing quality assurance or compliance exists | Comprehensive participation in system for assessing quality assurance or compliance |
|  | (b) Human resources | No or limited training implemented per national infection control standards | Staff trained in standards, skills, and leadership for infection control in all national hospitals | Staff trained in standards, skills, and leadership for infection control in most (>80%) sub-national hospitals | Plus staff trained in standards, skills, and leadership for infection control in most (>80%) hospitals one level below sub-national |
|  | (c) Logistical resources | No or limited availability of infection control materials; equipment is not available at central hospitals most of the time | Infection control materials generally available at national hospitals is sufficient to meet national infection control standards | Infection control materials generally available at sub- national hospitals is sufficient to meet WHO infection control standards | Infection control materials available in-house or at regional level with distribution capability to hospitals one level below sub-national sufficient to meet WHO infection control standards |
|  | (d) Institutionalization of infection control efforts | No or limited focus on infection control in governmental or private sectors | Unit within Ministry of Health with infection control as a major area of responsibility | Infection control committee or organizing body at each regional (between national and sub-national), sub- national, and hospital levels; data collected and reported | ≥1 active professional society principally committed to infection control; national plan exists to improve infection control within 3 years |
| Capability 12: Health sector pandemic response | (a) Surge capacity – human resources | No or limited planning for increasing human resources during pandemic phases 4-6; number of staff unknown | Formal plan for increasing human resources exists, and includes current capacity and projection of needs based on data; roster of staff and identification of extra staff complete | Budget established for procurement of resources and implementation of plan; software used to project needs | Plan and resources meet ≥50% of projected surge capacity needs; plan addresses legal issues |
|  | (b) Surge capacity – physical facilities and equipment | Number of beds unknown; no or limited planning to increase number of beds or home care | Formal plan to increase bed capacity or home care exists, and includes current capacity and projection of needs based on data | Budget established for procurement of resources and implementation of plan; software used to project needs | Plan and resources meet ≥50% of projected surge capacity needs |
|  |  | Number of ventilators and operators unknown; no or limited planning to increase ventilator and operator capacity | Formal plan to increase ventilator and operator capacity, and includes current capacity and projection of needs based on data | Budget established for procurement of resources and implementation of plan; software used to project needs | Plan and resources meet ≥50% of projected surge capacity needs |
|  | (c) Clinical management guidelines | No or limited clinical management guidelines for care of patients with suspect or novel strains of influenza | Clinical management guidelines exist and are widely available; guidelines meet or exceed WHO guidelines | Staff at national level trained in clinical management guidelines | Staff at sub-national levels trained in clinical management guidelines |
|  | (d) Surge capacity – care of deceased | No or limited planning for care of deceased | Plan for care of deceased exists, and includes current capacity and projection of needs based on data | Budget established for procurement of resources and implementation of plan; software used to project needs | Plan and resources for retrieval, identification, temporary storage, and culturally appropriate disposal of bodies meets ≥50% of projections |
